# Latent autoimmune diabetes in adults in China

**DOI:** 10.3389/fimmu.2022.977413

**Published:** 2022-08-25

**Authors:** Junlin Qiu, Zilin Xiao, Ziwei Zhang, Shuoming Luo, Zhiguang Zhou

**Affiliations:** Department of Metabolism and Endocrinology, National Clinical Research Center for Metabolic Diseases, Key Laboratory of Diabetes Immunology (Central South University), Ministry of Education, The Second Xiangya Hospital of Central South University, Changsha, China

**Keywords:** LADA, autoantibodies, epidemiology, genetics, HLA, Chinese

## Abstract

Latent autoimmune diabetes in adults (LADA) is a type of diabetes caused by slow progression of autoimmune damage to pancreatic beta cells. According to the etiological classification, LADA should belong to the autoimmune subtype of type 1 diabetes (T1D). Previous studies have found general immune genetic effects associated with LADA, but there are also some racial differences. Multicenter studies have been conducted in different countries worldwide, but it is still unclear how the Chinese and Caucasian populations differ. The epidemiology and phenotypic characteristics of LADA may vary between Caucasian and Chinese diabetic patients as lifestyle, food habits, and body mass index differ between these two populations. The prevalence of LADA in China has reached a high level compared to other countries. The prevalence of LADA in China has reached a high level compared to other countries, and the number of patients with LADA ranks first in the world. Previous studies have found general immune genetic effects associated with LADA, but some racial differences also exist. The prevalence of LADA among newly diagnosed type 2 diabetes patients over the age of 30 years in China is 5.9%, and LADA patients account for 65% of the newly diagnosed T1D patients in the country. As a country with a large population, China has many people with LADA. A summary and analysis of these studies will enhance further understanding of LADA in China. In addition, comparing the similarities and differences between the Chinese and the Caucasian population from the perspectives of epidemiology, clinical, immunology and genetics will help to improve the understanding of LADA, and then promote LADA studies in individual populations.

## Introduction

Latent autoimmune diabetes in adults (LADA) is a subtype of diabetes that belongs to autoimmune type 1 diabetes (T1D). Adult-onset diabetes (> 30 years at diagnosis), presence of diabetes-associated autoantibodies, and absence of the requirement of insulin requirement for at least 6 months after diagnosis are the key current diagnostic criteria for LADA from the insight of an international expert panel. The key clinical features of LADA and the requirement for no insulin at diagnosis mean that LADA shares similarities with type 2 diabetes (T2D). LADA refers to a type of diabetes that is characterized by slowly progressing autoimmune damage to islet beta cells in the early clinical stages without the need for insulin therapy. Differences between LADA and classic T1D/T2D in terms of genetic background, autoimmune response, rate of pancreatic islet function decline, and clinical metabolic characteristics have been found. Pathogenesis and clinical manifestations of LADA are highly heterogeneous, which has attracted the attention of diabetes experts, scholars, and clinicians. The classification, diagnostic criteria, and treatment of LADA are controversial. In 2019, the World Health Organization (WHO) classified LADA as mixed diabetes and considered LADA to be an independent type of diabetes (1), while the American Diabetes Association (ADA) classified it as a subtype of T1D (2). Based on the etiology of LADA, the new Chinese consensus suggests that LADA should be classified as a slowly progressing subtype of autoimmune T1D (3).

Numerous studies have been conducted to explore the incidence, immunogenetics, and clinical features of LADA. Some studies have been conducted in European countries including Italy (4, 5), Norway (6), the United Kingdom (7–9), Poland (10), Czech Republic (11), Finland (12), and Sweden (13), and in American regions including the United States (9), and Cuba (14), Studies have also been done in African countries including Madagascar (15), in Oceania including Australia (16), and in Asia including North India (17), South Korea (18), Japan (19, 20), Sri Lanka (21), United Arab Emirates (22), and Southeast Iran (23).

As one of the most populous countries in the world, LADA research in China has always attracted attention. In this review, the research progress concerning LADA in China in terms of epidemiology, genetics, immunology, clinical characteristics, diagnosis and classification, and treatment management is summarized based on similarities and differences compared to Caucasoid populations to promote further research of LADA. Some of the key comparison results are shown in **Table 1**.

**Table 1:** Comparison of latent autoimmune diabetes in adults between Chinese and Caucasian populations.

LADA	Chinese	Caucasian
**Epidemiology**		
LADA proportion of newly diagnosed phenotype T2D [Age ≥ 30 years, GADA(Only) %]	5.9% (24)	3.7% to 4.7% (4, 9)
LADA proportion of newly diagnosed phenotype T1D[Age ≥ 30 years, GADA (Only) %]	65% (25)	NA
High GADA titer LADA proportion in LADA	1/4 (24)	1/2 (4)
**Genetic characteristics**		
Family history of diabetes	25% (24, 25)	43%~ 66% (26, 27)
HLA-II susceptibility alleles	classic T1D> LADA>T2D (24, 28)	classic T1D> LADA>T2D (12, 29)
HLA II susceptibility haplotypes	DQA1*05-DQB1*0201、DQA1*03-DQB1*0401 and DQA1*03-DQB1*0303 (30)	DRB1*03-DQB1*0201 and DRB1*04-DQB1*0302 (30)
HLA-II susceptibility genotypes	DR9/DR9 (31)	DR3/DR4 (31)
PTPN22 gene	-1123G>C promoter polymorphism of PTPN22 (32)	+1858T variants of PTPN22 (33)
TCF7L2 single nucleotide polymorphism	NA	rs7903146 (34)
GWAS	NA -	HLA-DQB1gen rs9273368 (35)
**Islet autoantibodies**		
LADA patients		
Positive proportion of GADA	74.6% (36)	90.5% (8),96% (37)
Positive proportion of IA-2A	22.7% (36)	24.1% (8)
Positive proportion of ZnT8A	23.1% (36)	18.4% (8)
Positive proportion of Tspan7A	21.4% (38)	NA
Positive proportion of SOX13	13.1% (39)	7.0% (40)
Positive proportion of CPH-A	8.1% (41)	NA
Adults with diabetes		
Positive proportion of GADA	5.78% (42)	8.8% (8)
Positive proportion of IA-2A	1.51% (42)	2.3% (8)
Positive proportion of ZnT8A	1.84% (42)	1.8% (8)
Positive proportion of IAA	1.26% (42)	NA
**Clinical features of LADA**		
The proportion of patients with Metabolic syndrome	44.3%-50% (43, 44)	41.9-74.1% (9, 45, 46)
Insulin resistance	LADA=T2D (47)	LADA<T2D (9)
Glycemic variability	LADA>T2D (48)	NA
TPOAb	16.3-18.5% (49–51)	22.1- 41.7% (5, 10, 11, 27, 52–54)
TG-Ab	6.7%-16.3% (50, 51)	8.8%-28.6% (10, 11)
tTG-Ab	2.1% (49)	2%-2.9% (10, 27, 53)
21OH-Ab	1.8% (49)	1.7%-4.5% (10, 52, 53)
The proportion of patients with osteoporosis	T1D>LADA>T2D (55)	NA
The prevalence of microvascular complications	LADA<T2D (56)	LADA<T2D (57)
The prevalence of diabetic neuropathy (duration < 5 years)	LADA<T2D (56)	LADA<T2D (58)
The prevalence of macrovascular complication	LADA=T2D (57)	LADA=T2D (46, 57, 59, 60)
The prevalence of coronary heart disease	LADA=T1D (61)	LADA>T1D (62), LADA<T1D (63)
The prevalence of hypogonadism	LADA=T2D (64)	NA
**Treatment for islet beta cell protection**		
Insulin	Effective (65)	Effective (66)
dipeptidyl peptidase-4 inhibitor	Effective (65, 67)	Effective (68–71)
Rosiglitazone	Effective (72, 73)	NA
Vitamin D	(1α-hydroxy vitamin D3) Effective (74, 75)	(vitamin D3) Effective (71)
Glucagon-like peptide-1 receptor agonist	NA	Effective (64, 66)

NA, no applicable.

## Epidemiology of LADA in China

In China, two clinical studies have demonstrated that the estimated prevalence of LADA in patients with T2D is between 5.9% and 9.2% (24, 76). In the Chinese LADA study conducted by 46 centers in 25 cities across the country in 2006, the prevalence of LADA in newly diagnosed T2D patients over 30 years old was found to be 5.9% based on screening of a single glutamate decarboxylase autoantibody (GADA). A cross-sectional study in Tianjin (China) found that out of 8,109 participants, 498 (6.1%) were patients with T2D. Of these patients, 46 (9.2%) were found to have LADA. The prevalence of LADA was 0.6% (46 of 8,109). The Northern region has more patients than the Southern region, and a decreasing trend from the Northeast to the Southwest was found (24). However, single autoantibody screening found that LADA accounts for approximately 3.7% to 4.7% of the T2D in the Caucasian populations over 30 years (4, 9). After the age of the study subjects was restricted, the incidence of LADA in recently diagnosed patients with T2D in the Caucasian population was found to be lower than that in the Chinese population.

Similar to China, several large-scale epidemiological studies in Europe have also reported the prevalence of LADA with high levels in the North and low levels in the South (77). This finding is consistent with the geographic distribution trend of T1D incidence in children in China (78). Multi-center epidemiological data show that LADA patients account for 65% of new T1D patients in China (25). In our country, the use of combined multi-antibody screening can increase the LADA detection rate up to 8.6% (42). It is estimated that diabetes affected 113.9 million Chinese adults, with 79.6 million being newly diagnosed diabetes (79). If our rates of LADA in newly diagnosed diabetes are also applicable to other newly diagnosed diabetes in China, it can be estimated that China had 4.3 million adults with LADA (25). This huge population in China needs more research to explore the pathogenic mechanism of LADA in addition to effective diagnosis, treatment, and management.

## Genetic characteristics of LADA in China

The pathogenesis of LADA has a significant genetic background. Both T1D and T2D susceptibility genes are involved in the pathogenesis of LADA (13, 80). LADA patients in both the Chinese and Caucasian populations have family histories of diabetes, and the proportion in the Caucasian population ranges from 43% to 66% (26, 27), which is higher than in the Chinese population at 25% (24, 25). The loci most strongly associated with genetic susceptibility to LADA are the human leukocyte antigen (HLA) genes, especially HLA class II genes (81).

Studies in both Caucasian and Chinese populations have found that the frequency of HLA-II susceptibility alleles DR3, DR4, DQ2, and DQ8 in descending order are classic T1D, LADA, T2D, and healthy controls (12, 24, 28, 29). Racial differences in the HLA susceptibility genotypes of LADA exist. The susceptibility genotype of LADA patients in the Caucasian population is DR3/DR4, while the most common HLA-II susceptibility genotype in Chinese LADA patients is DR9/DR9 (31). The pooled results demonstrate that in China, DQA1*05-DQB1*0201, DQA1*03-DQB1*0401, and DQA1*03-DQB1*0303 were statistically significantly associated with increasing the risk of LADA (P < 0.001), while DQA1*0102-DQB1*0602 statistically significantly correlated with decreasing susceptibility to the disease (P = 0.003). As for Caucasian populations, both DRB1*03-DQB1*0201 and DRB1*04-DQB1*0302 were predisposed to the statistically significant development (P < 0.001) of LADA (30).

Genome-wide association studies (GWAS) of the Caucasian people have confirmed that most of the genetic characteristics of LADA are similar to those of classic T1D, and the most susceptible locus of LADA is the HLA-DQB1 gene rs9273368 (35). However, the frequency of HLA susceptibility genes in LADA is lower than that in classic T1D. A non-HLA genes related to GADA, the -1123G>C promoter polymorphism of the protein tyrosine phosphatase non-receptor type 22 gene (PTPN22), was associated with LADA in the Chinese population (32), and C1858T of PTPN22 was associated with LADA in the Caucasian population (33). GWAS of Caucasians have confirmed that non-HLA genes related to GADA, including the insulin gene (INS), cytotoxic T lymphocyte-associated protein 4 gene (CTLA4), SH2B adaptor protein 3 gene (SH2B3), and others are related to LADA (35). In recent years, LADA patients with rapid progression to insulin dependence have been identified using a genetic risk score model (82). Due to genetic heterogeneity among ethnic groups, in terms of these LADA studies, Chinese study is currently missing but is a future research direction, especially since the prediction of LADA genetic risk in the Chinese population needs a large-sample LADA genome-wide study

LADA is also associated with T2D susceptibility genes. Transcription factor 7 analog 2 (TCF7L2) is the most studied T2D susceptibility gene in LADA patients. The T2D-associated TCF7L2 single nucleotide polymorphism (SNP) in Chinese T2D subjects is the rs290487Callele (83), which is different from that in European T2D subjects, namely the rs7903146Tallele (84). A previous meta-analysis showed that the rs7903146 locus of TCF7L2 was a susceptibility gene in LADA patients in the European Caucasian population, especially in overweight patients (34). Other T2DM susceptibility genes, ZMIZ1 and KCNQ1, have also been found to be associated with LADA (13). However, the expression of TCF7L2, ZMIZ1, and KCNQ1 in the Chinese LADA population remains unclear.

## Immunological features of LADA in China

LADA is a T-cell-mediated autoimmune disease, which belongs to autoimmune-mediated diabetes from the perspective of etiology and pathogenesis. The abnormal humoral immunity of LADA is mainly manifested by the presence of islet autoantibodies in the serum of patients. Common islet autoantibodies in T1D clinical practice include GADA, insulin, protein tyrosine phosphatase, and zinc transporter-8 antibodies (GADA, IAA, IA-2A, and ZnT8A, respectively). Among them, GADA is the most common islet autoantibody in LADA patients. The positive rate of GADA is significantly higher than that of other antibodies, such as IA-2A, ZnT8A, and IAA (38, 49, 85). A Chinese LADA study that included 3062 newly diagnosed T2D patients found that the positive rates of GADA, IA-2A, and ZnT8A were 6.43%, 1.96%, and 1.99%, respectively (85). A nationwide survey based in China showed that among the 264 LADA patients, the positive rates of GADA, IA-2A, and ZnT8A were 74.6%, 22.7%, and 23.1%, respectively (36). In the European Action LADA study, 90% of adult-onset diabetic patients had GADA, while IA-2A and ZnT8A only were responsible for 10% of the remaining cases (8). Therefore, the diagnostic value of GADA antibodies in Caucasian LADA is better than that in the Chinese population. Chinese LADA screening is often based on GADA combined with IA-2A, ZnT8A, and IAA detection to improve diagnostic sensitivity. In addition, Chinese LADA may also have transmembrane protein 7 (Tspan) autoantibodies (38), carboxypeptidase H (CPH-A) autoantibodies (41), and transcription factor Syr-Box transcription factor (SOX13) antibodies (39). SOX13-A was found in 7.0% of Caucasian individuals with LADA (40), which was lower than that found in the Chinese (39). For the other two antibodies, no results from studies in the Caucasian population are currently available. The new islet autoantibody Tspan7A, with a positive rate of 21.4% in LADA patients, can predict LADA islet failure; thus, it is a promising new immune marker (38). At present, research on the autoantibodies of LADA in China is ongoing, and it is expected that there will be better indicators for diagnosis in the future will be found.

Cellular immunoblotting technology was used to show that some patients with clinically diagnosed T2D who were negative for antibodies had a cellular immune response to islet proteins (86–88). Such LADA patients are called “T-LADA” in spite of the lack of islet antibodies (89). Their peripheral blood T-cells show an immune response to islet antigens (89), and their islet failure occurs more rapidly than that of T2D patients (90). Studies in China have also confirmed the existence of T-LADA (91). The enzyme-linked immuno-spot (ELISPOT) assay showed that GAD65-reactive T-cells exist in LADA (92), suggesting that the combination of T-cells and islet autoantibody detection can improve the diagnostic sensitivity (93, 94). In a Chinese population study, the number of interferon-gamma (IFN-γ)-producing T-helper 1 (Th1) cells increased in LADA patients when compared with T2D patients after islet antigen stimulation. The ratio of Th1/Th2 T cells also increased in LADA after this process (92). In addition, another Chinese study showed that T-cells analyzed at the mRNA and cellular levels showed a reduction in regulatory T-cells (T-regs) in LADA compared to T2D (95). The adoptive transfer regulatory T-cells seem to be potential therapeutic targets for LADA. The abnormal cellular immunity of LADA is mainly manifested by changes in the number and function of various immune cells and their subgroups in the blood circulation. T-regs inhibit the activation of pathogenic T-cells, thereby inducing immune tolerance. The increased methylation of the forkhead box p3 (Foxp3) promoter region in CD4+ T cells in LADA patients of Chinese can lead to a decrease in T-reg numbers and functional defects and induce LADA autoimmunity (96, 97). Studies of Caucasians have shown that the distribution of T-cell subsets correlates with C-peptide levels in LADA patients, suggesting that it may be used to predict changes in LADA islet functions (98).

The study found that in addition to T-cells, the frequencies of peripheral regulatory B-cells (B-regs), marginal zone B-cells (MZB), and follicular B-cells (FOB) in LADA patients had significantly changed when compared with those in healthy people, suggesting that B-cells may be involved in the pathological mechanism of LADA (99). Studies in both the Chinese (100) and Caucasian (99) populations have shown that the proportion of B-regs in LADA patients is higher than in T1D patients.

Natural killer (NK) cells and neutrophils play important roles in innate immunity-mediated local immune responses and linked adaptive immune responses. The number of peripheral NK cells in the Caucasian who have LADA diagnosed within 5 years is reduced, and the spectrum of NK cell subsets is altered compared to normal NK cells (101). In contrast, newly diagnosed Caucasian patients with LADA showed a higher proportion of NK cells than both healthy controls and patients with T2D (99). In a Chinese population, it was found that patients diagnosed with LADA within 1 month had a higher frequency of CD3^−^CD56^+^ NK cells, activated NKp46^+^ NK cells, and IFN-γ^+^ NK cells than healthy control individuals. Moreover, the percentages of circulating NKp46^+^NK cells were negatively correlated with the levels of fasting plasma C-peptide (102). We recently found higher numbers of neutrophils in the circulation of newly diagnosed LADA patients (diagnosed within 1 year) compared to patients with type 1 diabetes. The neutrophil counts in patients with LADA were negatively correlated with the levels of GAD, IA2, and ZnT8 autoantibodies (103). The RNA expression profile of neutrophils in Chinese LADA patients was different from that in healthy controls (104), suggesting that innate immunity is also involved in the pathogenesis of LADA. However, in patients with Caucasian LADA, Singh, et al. found no alteration in the proportions of CD15^low^ neutrophils in the patients with a mean disease duration of close to 5 years (99). Caucasian studies have also showed that dendritic cells (DCs) (99) and Monocytes (105) also are related to the pathogenesis of LADA, but a lack of research evidence in the Chinese population exists.

Further studies showed that blood levels of inflammatory cytokines in Caucasian LADA patients are similar to those in T1D subjects, including interleukin (IL)-6 and tumor necrosis factor (TNF)-a (106). However, we previously found that subjects with LADA had distinct cytokine profiles compared with subjects with type 2 diabetes and type 1 diabetes regarding lipocalin 2 (LCN2), adiponectin, IL-6, and high-sensitivity C-reactive protein (hs-CRP) (107).

**Figure 1:**
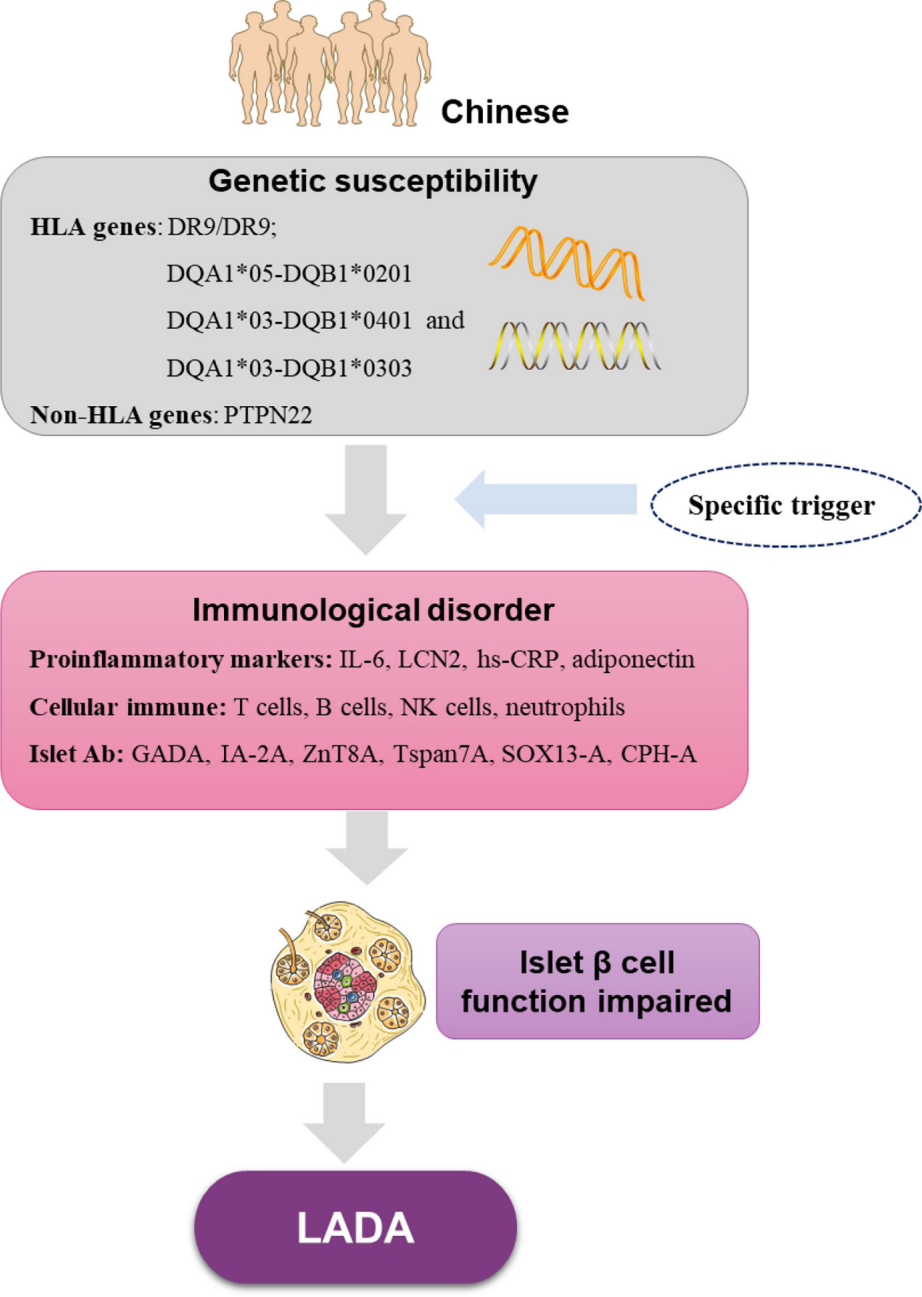
The immunological features and genetic characteristics of Chinese LADA base on current research. HLA, human leukocyte antigen genes; PTPN22, protein tyrosine phosphatase nonreceptor 22; IL-6, interleukin-6; LCN2, lipocalin 2; hs-CRP, high-sensitivity C-reactive protein; NK cells, natural killer cells; GADA, glutamic acid decarboxylase autoantibody; IA-2A, protein tyrosine phosphatase IA-2; ZnT8A, zinc transporter-8 antibodies; Tspan7A, transmembrane protein 7 autoantibodies; CPH-A, carboxypeptidase H autoantibodies; SOX13-A, Syr-Box transcription factor antibodies.

Recently, a Caucasian study detected TNF-α and IL-1β gene expression in the pancreata of patients and rats with LADA, and found a shift in the proinflammatory cytokine gene expression from TNF-α to IL-1β in the pancreata from LADA compared to that from T1D subjects (108). Additionally, other cytokines, such as IL-6 and IL-15, may also contribute to LADA pathogenesis. A Caucasian study demonstrated that the circulating IL-6 and -15 concentrations are significantly higher in both patients with LADA and their healthy first-degree relatives compared to healthy control subjects, supporting a role for pro-inflammatory role of this cytokine in mediating susceptibility to LADA (109). Chinese study confirms that the circulating IL-6 concentration is significantly higher in both patients with LADA compared to healthy control subjects (107). However, the study of cytokines in pancreatic tissue of LADA patients and first-degree relatives of LADA patients is still uncertain. In addition, in order to more clearly illustrate the immunological and genetic characteristics of LADA in China, we made **Figure 1** to show it.

## Clinical manifestations of LADA in China

The clinical course of LADA can be divided into non-insulin- and insulin-dependent stages (7). In the non-insulin-dependent stage, LADA occurs early in the clinical stage, and the patient’s performance is similar to that of T2D patients with no typical symptoms of hyperglycemia and no tendency toward spontaneous ketosis. Oral hypoglycemic drug treatment can control blood glucose in this situation. At this stage, islet β cells not only function abnormally but also start to decrease in number, which is a key factor in disease progression (47). Islet β-cell function decline in LADA is more rapid than in T2D but slower than in classic T1D. Among them, la ow titer of GADA was found to be a predictor of β-cell function retention in LADA patients (110), and the pattern of decreasing islet β-cell function in LADA was found to show a biphasic pattern of rapid followed by a slow decline. The declining pattern in C-peptide was rapidly followed by a slow biphasic mode with about one-quarter of LADA patients developing beta-cell function failure during the first 8 years (110). A GADA titer < 173.5 units/mL was found to be indicative of beta-cell function preservation. When the patient’s pancreatic β-cell function is significantly insufficient and in diabetic ketosis or acidosis results, insulin treatment is necessary, and the insulin-dependent stage begins. The decrease in the rate of C-peptide in LADA patients in China is three times that of T2D (111). The time point of the progression from insulin independence to insulin dependence is highly heterogeneous and appears to be related to age at onset, antibody titer, and multiple islet antibody positivities (7, 112); The GADA titer is the strongest predictor of islet β-cell failure in LADA patients index, which has been confirmed in both Chinese (110) and Caucasian (4) populations.

In individuals with adult-onset diabetes, the presence of N-terminally truncated GAD65 autoantibodies is associated with the clinical phenotype of autoimmune T1D and predicts insulin therapy. Studies of LADA have shown that targeting the N-terminal epitope GADA, rather than the full-length GADA, is better for predicting the progression of LADA to insulin-dependent therapy (113). However, no studies addressing Chinese LADA patients are available. Chinese patients with high GADA titers and reactivities to GADA65 middle and C-terminal regions showed persistent GADA positivity in which a poor baseline and accelerated decline of β-cell function need early intervention in clinical practice (114).

The clinical phenotype of GADA-positive LADA is most common: these patients are younger, thinner, have poorer beta-cell function, and have a faster rate of islet decay than other patients who are positive for only one antibody. The islet function of Tspan7A-positive LADA decreased more rapidly than that of negative LADA (38). Although GADA-positive LADA patients with IA-2A and/or ZnT8A positive are rare, they have a younger clinical phenotype and a more acute and faster decline in islet function than GADA-positive patients alone, which has been confirmed in both Caucasian and Chinese populations (115–117). LADA can be divided into two subtypes according to the level of GADA titer (118) : (1) LADA with high-titer has similar clinical characteristics to classic T1D with a faster rate of islet function decline and less metabolic syndrome and (2) low-titer LADA is similar to T2D (24, 50). The ratio of LADA with high- to low- titer LADA in Chinese patients was reported to be about 1:3 (24), while in the Caucasian population it was 1:1 (4).In addition, blood glucose fluctuations of LADA patients were more acute than those of T2D (48). A Caucasian population study shows that individuals with LADA have worse glycemic control than patients with T2D despite a longer duration of insulin therapy (119).

In LADA, body mass index (BMI), waist circumference, waist-to-hip ratio (WHR), blood pressure, and triglyceride levels were lower than those in T2D (24, 45). Studies have shown that being overweight or obese is also a risk factor for LADA, especially in those with a family history of diabetes (120). The proportion of LADA patients with metabolic syndrome in China was found to be slightly lower than those with T2D but higher than T1D and healthy controls (24, 43). Metabolic syndrome findings in the Caucasian LADA population were similar to the Chinese population, but the Caucasian population had a higher proportion of metabolic syndrome than the Chinese (45). Metabolic characteristics of LADA may vary with the age of onset. When compared with T2D patients with young-onset diabetes, elderly LADA patients (≥ 60 years of age at onset) had better islet β-cell function, more severe insulin resistance, and higher proportions of patients with metabolic syndrome, whose metabolic characteristics were similar to those of elderly T2D (121). Chinese studies have shown that gut microbiota and metabolite profiles are significantly different from those of healthy subjects and the authors also found a correlation among the gut microbiota, fecal and serum metabolites, and clinical phenotypes (58). Among men with LADA in the Caucasian population, leptin was positively and significantly correlated with BMI and fat mass. A Cox regression analysis showed that leptin levels were inversely and significantly related to the risk of early insulin dependence. Higher leptin secretion may exert a direct effect on beta cell function thus leading to more insulin sensitivity (122). However, there is no leptin study evidence from Chinese LADA patients is available.

Racial differences in insulin resistance in LADA have been found. A Chinese study used a hyperinsulinemic positive glucose clamp to evaluate insulin sensitivity and showed that the degree of insulin resistance in LADA was similar to that in T2D patients (47). In the Caucasian population, LADA patients present less insulin resistance than those with T2D (9). Insulin resistance caused by an imbalance of inflammatory cytokines in the blood circulation of LADA may be an important mechanism for the occurrence of metabolic syndrome (107, 123). Studies of Caucasians showed plasma adiponectin levels were higher in LADA patients when compared with controls. In LADA patients, plasma adiponectin levels, after adjustment for BMI, correlated significantly with insulin resistance (124). Studies in the Chinese population also showed plasma adiponectin levels were higher in LADA patients when compared with controls (107).

In addition, studies in both Chinese and Caucasian populations showed that the bone mineral density (BMD) of LADA patients was between T1D and T2D patients (55, 125). Studies in the Chinese population also showed that the proportion of patients with osteoporosis in the T1D, LADA, T2D, and control groups was 55.6%, 45.4%, 34.3%, and 26.9%, respectively (55). Another Chinese LADA study with males also showed that the proportion of osteoporosis in LADA patients was higher than that in T2D patients (126).

The proportion of chronic complications in LADA patients differs from that in T2D patients. Chinese population studies show that in the early stage of diabetes (duration < 5 years), the prevalence of retinal and renal diseases in LADA were lower than that in T2D (57). Similarly, Baum et al. reported that LADA patients with short-term diabetes (duration < 5 years) had fewer features of diabetic neuropathy than T2D patients in the early stages of disease; thus, the LADA resembled classical T1D patients who normally develop diabetic neuropathy rather late in the course of their diabetes (127). Decision tree analysis showed that in patients with a duration of diabetes of at least 10.5 years, T1D and LADA patients had a higher incidence (72.7% versus 55.1%) of diabetic retinopathy (DR) than T2D patients (123). Another follow-up study, mostly with Caucasians, also showed that at diabetes onset, LADA patients had a lower risk of microvascular complications followed by a delayed higher risk of complications than adults with T2D, secondary to worse glycemic control. Implementing strict glycemic control from the time of diagnosis could reduce the later risk of microvascular complications in adults with LADA (56).

Abnormal nerve conduction was found to be present in 18.5%, 38.8%, and 66.7% of LADA patients with duration of < 5 years, 5–14 years, and ≥ 15 years, respectively. LADA patients had higher rates of diabetic neuropathy than T2D patients over the disease course of 5 to 14 years. Peripheral nerve dysfunction is common in asymptomatic patients with LADA or T2D. Study findings suggest that LADA and T2D differ in the pattern of peripheral nerve involvement over diabetes duration (128). In another study of diabetic neuropathy in a Chinese population, the proportion of diabetic neuropathy was higher in T2D patients (42.3%) than in LADA (23.6%), but their disease courses were not matched (129). Another study from Hong Kong showed that the proportion of diabetic neuropathy in LADA patients did not differ from that in patients with T2D, but the study mixed patients with early and advanced diabetes, so the results need to be further analyzed (61). The proportion of diabetic neuropathy in LADA was higher than that in T2D in the Caucasian population, but the proportions were not statistically significant (59, 62). Despite comparable age and duration of diabetes, participants with LADA demonstrate more severe neuropathy and particularly small fiber neuropathy, when compared with participants with T2D (130). The different results involving neuropathy are partly due to the different criteria for neuropathy. It is still necessary to match the course of the disease, age, and diagnostic criteria before more accurate conclusions can be drawn.

A comparison of cardiovascular disease (CVD) prevalence in LADA patients with T1D or T2D is controversial and may be related to differences in ethnicity, metabolic control, and disease duration. A retrospective medical record study from China showed that the proportion of CVD in T2D patients was lower than that in LADA but no statistically significant difference was found (129). In another study, the prevalence of carotid plaques and CVD was also comparable between patients with LADA and T2D patients regardless of diabetes duration (57). LADA patients had a lower risk of CVD when compared with T2D patients, No differences in the risk of CVD in patients with LADA when compared with T1D were found (61). Multiple Caucasian populations have shown that no significant difference in the incidence of macrovascular complications in patients with LADA and patients with T2D exists (46, 59, 60, 62). Caucasian LADA patients in Finland were found to have a higher incidence of CVD than T1D patients (62), whereas Caucasian LADA patients in Denmark had a lower incidence of CVD than T1D (63). Systemic concentrations of adhesion molecules in addition to chemokines are associated with an increased risk of cardiovascular complications. The LADA study of Caucasian investigated the soluble adhesion molecule as well as the chemokines, and found that the levels of the adhesion molecules and chemokines are similar in individuals with LADA and T1D (131). However, there is no similar study evidence from Chinese LADA patients is available.

LADA is sometimes associated with other autoimmune diseases or autoimmune-related antibodies. Common autoimmune diseases in Chinese LADA patients include autoimmune thyroid disease, celiac disease, Addison’s disease, and autoimmune gastritis (49, 50). The positive rates of thyroid peroxidase antibody (TPO-Ab), celiac disease-related transglutaminase antibody (tTG-Ab), and Addison’s disease-related 21-hydroxylase antibody (21OH-Ab) in Chinese LADA patients were 16.3%, 2.1% and 1.8%, respectively (49). LADA is most often associated with thyroid antibody TPO-Ab and autoimmune thyroid disease (49); nearly 20% of LADA patients with high titers of GADA have autoimmune thyroid disease, and subclinical thyroid dysfunction is the most common thyroid problem (50). Several autoimmune-related antibodies were also found in LADA patients in the Caucasian population (52, 53), and the positive ratio of TPOAb (5, 52, 53) was higher than that in the Chinese population, a finding that may be related to the higher proportion of high-titer GADA. In addition, a Chinese study showed that the rate of hypogonadism in the LADA and T2D groups were 8.2%, and 21.7%, respectively (p = 0.017). After adjusting for possible confounders, the rate of hypogonadism in the LADA group was comparable to those of the T2D group (64).

Although different clinical and metabolic features of T1D, LADA, and T2D suggest that they have different pathophysiology and prevalence of chronic complications, few studies have investigated the risk of complications in Chinese LADA patients, especially prospectively or with different interventions. In addition, a lack of studies on acute complications in LADA patients exists.

## Treatment of LADA in China

The goal of LADA treatment is to achieve an ideal level of glucose metabolism control, regulate islet autoimmunity, protect islet β-cell function, and prevent diabetes complications and associated complications. Due to the biphasic pattern of islet β-cell dysfunction in Chinese LADA patients (110), it is recommended that patients with certain islet function should be followed to assess the level of C-peptide and GADA titer (132), and the treatment plan should be adjusted accordingly.

### Treatment backed by Chinese research evidence

At present, the drugs that have been preliminarily proven to have potential efficacy in the LADA population in China include insulin, dipeptidyl peptidase-4 inhibitors, thiazolidinediones (TZD) drugs, and vitamin D. However, these clinical studies were based on small sample size. More evidence is required in patients with LADA later.

Insulin therapy can protect islet β-cell function in LADA patients by promoting islet rest and inducing immune tolerance (133). Both Chinese and Caucasian populations have demonstrated that the time to progression of LADA to insulin dependence is related to GADA titers (110, 115). Chinese population studies have shown that insulin offers limited protection of islet function in LADA patients (65). For Chinese LADA patients with high GADA titers, multiple islet autoantibodies, low C-peptide levels, and/or poor glycemic control, early initiation of insulin therapy is suggested.

Dipeptidyl peptidase-4 inhibitor (DPP-4i) can inactivate the DPP-4 enzyme, lead to an increase in the level of GLP-1, promote insulin secretion from pancreatic β-cells, and lead to lower blood glucose A randomized controlled trial in a Chinese population found that insulin plus sitagliptin outperformed insulin alone to maintain C-peptide level (65, 67). Other studies involving the Caucasian population studies have also reported similar benefits of saxagliptin (68, 69) and linagliptin (70). When compared with insulin intervention alone, sitagliptin plus insulin treatment appears to maintain β-cell function and improve insulin sensitivity to some extent in LADA patients (69).

TZD drugs activate intracellular peroxisome proliferator-activated receptor (PPAR), lead to enhanced insulin sensitivity, and have anti-inflammatory and immunomodulatory effects. Rosiglitazone can produce improvements in pancreatic β -cell function in a Caucasian T2D population (134). This pilot study suggests that rosiglitazone combined with insulin may preserve islet β-cell function in LADA patients (72, 73). The use of TZD to treat LADA in the absence of drug contraindications is recommended, but paying close attention to side effects, such as edema, cardiac function, anemia, and fractures, is necessary.

Vitamin D plays an anti-inflammatory and immunomodulatory role *via* the vitamin D receptors (VDR). Functional VDRs exist in almost all immune cells, and the polymorphism of key genes of vitamin D metabolism is associated with T1D (135). A Chinese study has shown that the combination of 1-alpha-hydroxyvitamin D3 (1-alpha(OH)D3 and insulin when compared with insulin alone led to an improvement in fasting C-peptide levels in patients with LADA (74), Saxagliptin combined with vitamin D3 2000 IU/day showed protective effects on pancreatic β-cell function in LADA patients (75). A Brazilian study showed that sitagliptin combined with vitamin D3 5000 IU/day extended the mean honeymoon period to 27.1 months in patients with T1D (71).

### Other treatments

Drugs validated by clinical research evidence in the Chinese population are undoubted of great significance to support the use of the drug, but for some drugs that have corresponding results in the Caucasian population but not in the Chinese population, we also agree with the conclusions found in Caucasian populations. However, for some drugs for which no corresponding Chinese LADA population study data are available, their efficacy needs to be further evaluated.

Glucagon-like peptide-1 receptor agonist (GLP-1RA) acts on islet beta cells to promote insulin synthesis and secretion and acts on islet alpha cells to inhibit glucagon release. This peptide can inhibit appetite, slow stomach emptying, and lead to lower blood sugar levels. A *post hoc* analysis of dulaglutide trials in a Caucasian population showed that the hypoglycemic effect of GADA-positive subjects with dulaglutide was comparable to that of GADA-negative subjects (136). Analysis of GADA or IA-2A positive patients with newly diagnosed T2D using exenatide and liraglutide (137) showed that patients with low C-peptide levels (fasting C-peptide ≤ 0.25 nmol/L) had poor hypoglycemic effects. However, the effect on the Chinese LADA population is unclear.

Next, although no reports of metformin monotherapy for LADA are available, trials of combined treatment consisting of metformin other drugs can be found (68). No clinical research reports on the use of this drug in the Chinese population are available, but metformin is not only a first-line drug for T2D but also has indications for combined use with insulin in the treatment of T1D (138), so it is also recommended to use it in the Chinese population.

Concerning sulfonylureas, studies in the Caucasian population have shown that LADA patients treated with sulfonylureas progress to insulin dependence more quickly than when taking other drugs (66, 70, 115, 139). Although no clinical trials addressing sulfonylurea use in the Chinese population have been done, sulfonylureas cause more rapid LADA islet hypofunction, which may be related to its direct action on islet β-cells, promotion of insulin release, and acceleration of β-cell apoptosis (140). It is recommended that LADA patients in China avoid sulfonylureas.

The sodium-glucose co-transporter 2 inhibitor (SGLT2i) causes a reduction in blood glucose by inhibiting the proximal sodium-glucose co-transporter in the renal tubules that promotes urinary glucose excretion. This class of drugs is the recommended class for diabetic patients with atherosclerotic cardiovascular disease (ASCVD) or those with high-risk factors for ASCVD, chronic kidney disease (CKD), and/or heart failure (141, 142). Although no research report on the treatment of LADA with such drugs has been published, dapagliflozin and sotagliflozin have been approved in the European Union for the treatment of adult T1DM patients with poor blood sugar control and BMI > 27Kg/m^2^ who are receiving insulin therapy (143, 144). These drugs are recommended for patients with T1D and have good effects in patients with heart failure. These drugs are also recommended for Chinese patients, but it is still necessary to verify its effect on LADA β-cell function in the Chinese population.

The islet-specific antigen, the GAD65 vaccine, helps protect islet β-cell function in LADA patients (145), especially in those with low GADA titers, but it remains to be verified by expanded studies. However, the use of this vaccine has not been carried out in the Chinese population, and whether it is effective in the Chinese population still needs to be confirmed.

Moreover, management of LADA published in Diabetes in 2020 advocated that C-peptide measurement should drive the decision-making process for the choice of LADA treatment. The panel proposed three broad categories of C-peptide levels: C-peptide levels < 0.3 nmol/L, C-peptide levels ≥0.3 and ≤ 0.7 nmol/L, and C-peptide levels > 0.7 nmol/L (132). Our Chinese LADA consensus recommends the following LADA treatment management process: firstly, according to the C-peptide level, then according to the GADA titer and whether it is complicated with cardio-renal diseases, select the corresponding drug for treatment (3). The Chinese consensus and international consensus are different, we also added antibody titer measurement to drive the decision-making process for the choice of LADA treatment. But these consensuses are undoubtedly a good start for LADA treatment. It is foreseeable that LADA patients will be more standardized in the future, and more research and evidence to support the treatment of patients will be available.

## Conclusions

In this review, LADA-related studies in China are summarized and compared with data from the Caucasian population. We hope that summarizing the research concerning Chinese LADA patients combined with the research from the Caucasian population will form the future direction of Chinese LADA research, such as the prediction of LADA genetic risk in the Chinese population. Although the different clinical and metabolic features of T1D, LADA, and T2D suggest that they have different pathophysiological characteristics and prevalence of chronic complications, few studies have investigated the risk of complications in Chinese LADA patients, especially prospectively or with different interventions. In addition, a lack of studies addressing acute complications in LADA patients exists. Regarding the treatment of LADA, some drugs have been studied in the Chinese population (insulin, dipeptidyl peptidase-4 inhibitors, TZD drugs, and vitamin D), and some drugs have only been studied in the Caucasian population (GLP-1RA, sulfonylureas, islet-specific antigen GAD65 vaccine). It is hoped that all promising drugs can eventually yield data on the Chinese population so that clinicians can be more evidence-based and accurate when treating LADA patients in China. The research that has been carried out in by the Chinese population is also expected to be supported by the Caucasian population, which will enable the establishment of effective methods to predict, prevent, and intervene in LADA in the future.

## Author contributions

JQ and ZX written the first draft of the manuscript. ZZW performed the material preparation and data collection. SL and ZZ reviewed manuscript and contributed to discussion. SL and ZZ proposed the project and are the guarantors of this work and, as such, had full access to all the data in the study and takes responsibility for the integrity of the data and the accuracy of the data analysis. All authors contributed to the article and approved the submitted version.

## Funding

This study was supported by the Hunan Province Natural Science Foundation in China (Grant No. 2020JJ2053), postgraduate Research Innovation Project of Hunan Province (CX20210367), and independent exploration and innovation projects for postgraduate of Central South University (Grant No. 2021zzts0365).

## Conflict of interest

The authors declare that the research was conducted in the absence of any commercial or financial relationships that could be construed as a potential conflict of interest.

## Publisher’s note

All claims expressed in this article are solely those of the authors and do not necessarily represent those of their affiliated organizations, or those of the publisher, the editors and the reviewers. Any product that may be evaluated in this article, or claim that may be made by its manufacturer, is not guaranteed or endorsed by the publisher.
